# Viral and serological kinetics in Zika virus-infected patients in South Korea

**DOI:** 10.1186/s12985-017-0740-6

**Published:** 2017-04-07

**Authors:** Young Eui Jeong, Go-Woon Cha, Jung Eun Cho, Eun Ju Lee, Youngmee Jee, Won-Ja Lee

**Affiliations:** 1 Division of Arboviruses, National Institute of Health, Korea Centers for Disease Control and Prevention, 187 Osongsaengmyeong 2-ro, Osong-yeup, Cheongju-si, Chungbuk-do 28159 South Korea; 2 Korea Centers for Disease Control and Prevention, Cheongju-si, 28159 Chungbuk-do South Korea

**Keywords:** Zika virus, Chikungunya virus, Dengue virus, Reverse transcription-polymerase chain reaction, Enzyme-Linked Immunosorbent Assay, Phylogenetic analysis

## Abstract

Zika virus is a mosquito-borne flavivirus that causes clinical symptoms similar to those observed in dengue and chikungunya virus infections. The Korea Centers for Disease Control and Prevention initiated laboratory testing using a real-time reverse transcription-polymerase chain reaction in January 2016. More than 1,000 suspected cases of infection were tested and nine were confirmed as imported cases of Zika virus infection from January to July 2016. The travel destinations of the infected individuals were Brazil, Philippines, Viet Nam, Guatemala, Puerto Rico, and the Dominican Republic. Phylogenetic analysis based on the partial envelope gene indicated that the viruses belonged to the Asian genotype circulating in South America. We further investigated the duration for which the viral RNA and virus-specific antibodies were detectable after the symptom onset. After the day of symptom onset, Zika virus was detectable until 6 days in serum, 14 days in urine and saliva, and 58 days in semen. Immunoglobulin M against Zika virus was detected as early as 2 days after the symptom onset and was maintained at these levels until 41 days, whereas Immunoglobulin G was detectable from 8 days after the symptom onset and was maintained until 52 days. These findings would help diagnostic laboratories improve their testing programs for Zika virus infection.

## Findings

Zika virus is a mosquito-borne virus belonging to the genus *Flavivirus* in the family *Flaviviridae.* The virus was first isolated from a sentinel monkey in the Zika forest in Africa in 1947 [1]. It causes mild symptoms such as fever, rash, myalgia, arthralgia, and retro-orbital eye pain, which are similar to those caused by dengue and chikungunya virus infection [2]. The transmission of the virus was limited to the African continent until 2007, when an outbreak occurred in Yap Island of Micronesia [3]. Since then, the virus has disseminated rapidly to Asia and South America. In February 2016, the World Health Organization declared Zika virus infection associated with microcephaly as a Public Health Emergency of International Concern.

The Korea Centers for Disease Control and Prevention (KCDC) designated Zika virus infection as a notifiable disease and initiated laboratory testing for this disease in January 2016. All persons who had travelled to countries with active Zika virus transmission in the 2 weeks prior to the onset of symptoms were suspected to be infected with the Zika virus, and laboratory testing was recommended. Sexual partners of persons with suspected or confirmed Zika virus infection were also recommended to undergo laboratory testing. The KCDC used a custom-made real-time reverse transcription-polymerase chain reaction (qRT-PCR) kit, namely the genesig Zika Virus polyprotein standard kit (PrimerDesign Ltd., UK) for detection of viral RNA in various types of samples. To detect the virus, viral RNA was extracted from 140 μL of sample using the QIAamp Viral RNA Mini kit (QIAGEN, Hilden, Germany) according to the manufacturer’s instructions. No treatment, such as concentration or filtration of the sample, was done prior to RNA extraction. The contents of genesig Zika Virus polyprotein standard kit are as follows: virus specific primer/probe mixture, positive control template (in vitro-transcribed RNA transcript), nuclease-free water, and 2× qRT-PCR Mastermix. Sequences of the primers and probe were not disclosed according to the company’s policy. The qRT-PCR mixture consisted of 10 μL 2× qRT-PCR Mastermix, 1 μL primer/probe mixture, 4 μL nuclease-free water, and 5 μL RNA sample. Thermal cycling conditions for reverse transcription were as follows: 55 °C for 10 min, enzyme activation at 95 °C for 2 min followed by 45 cycles of PCR (95 °C for 10 s, 60 °C for 60 s). The amplification was performed using the ABI StepOne Plus or 7500 instrument (Applied Biosystems, Foster City, CA, USA). A sample with a threshold cycle (C_T_) number ≤ 40 was considered to be positive for Zika virus infection. We additionally performed qRT-PCR according to the protocol described by Lanciotti et al. (1086/1163/1108FAM set), as a reference method [3].

We conducted RT-PCR assay to obtain the DNA sequence of the partial envelope (E) gene from Zika-positive samples [4]. The composition of the reaction mixture (25 μL) was 5 μL 5× QIAGEN One Step RT-PCR buffer, 1 μL dNTP mix, 1.5 μL each of the forward (ENV_F, GCT GGD GCR GAC ACH GGR ACT; 10 μM stock) and reverse primers (ENV_R, RTC YAC YGC CAT YTG GRC TG; 10 μM stock), 1 μL One Step RT-PCR Enzyme Mix, 10 μL nuclease-free water, and 5 μL RNA sample. The thermal profile for RT-PCR was as follows: reverse transcription at 50 °C for 30 min, enzyme activation at 95 °C for 15 min followed by 40 cycles of PCR (94 °C for 20 s, 55 °C for 20 s, 72 °C for 30 s) and a final extension cycle at 72 °C for 3 min. The positive products (~365 base pair, bp) were analyzed by electrophoresis using 1.5% agarose gel, purified from the gels using the QIAquick gel extraction kit (QIAGEN), and sequenced using the ABI PRISM BigDye Terminator Cycle Sequencing kits and ABI 3730xl sequencer (Applied Biosystems). The resulting sequence files were compiled using the SeqMan program in the Lasergene software version 8.0 (DNASTAR, WI, USA) and final 340 bp of E gene sequences were obtained. The sequence divergence (p-distance) among the E gene sequences was calculated using the Distance menu of the MEGA program (version 6.06). For phylogenetic analysis, 23 E gene sequences of Zika virus deposited in the GenBank were selected and seven new sequences obtained in this study were added. A multiple alignment of the sequences was done by the ClustalW method in the MEGA software. Phylogenetic tree was constructed by neighbor-joining method using the substitution model of maximum composite likelihood. The reliability of the tree was assessed by bootstrap analysis (1,000 replicates).

We further investigated the duration for which the viral RNA was detectable in various specimens (serum, urine, saliva, and semen), as number of days after the symptom onset. Specimens were collected until 85 days after the symptom onset at 1-week intervals. We also conducted serological testing for serum samples to reveal the antibody kinetics in patients with Zika virus infection, using three commercial kits: Quantitative Human Zika Virus IgM ELISA kit (MyBioSource Inc., San Diego, CA, USA), Zika virus IgM/IgG Ab Rapid Test (Biocan Diagnostics Inc., Canada), and Anti-Zika Virus IgM/IgG ELISA (Euroimmun, Germany). Among the 3 kits, the performance of only Euroimmun ELISA kit (IgM sensitivity, 90.1%; IgG sensitivity, ~50%) was previously reported [5]. Furthermore, the serological cross-reactivity of Zika-positive serum with other flaviviruses, including dengue, Japanese encephalitis, and West Nile viruses, was investigated using DENV Detect IgM capture ELISA, JE Detect IgM capture ELISA, and West Nile Detect IgM capture ELISA (InBios, Seattle, WA, USA), respectively.

From January to July 2016, more than 1,000 suspected cases of Zika virus infection were tested and nine were confirmed as cases of imported Zika virus infection. The destinations of travel were Brazil (1 case), Philippines (3 cases), Viet Nam (2 cases), Guatemala (1 case), Puerto Rico (1 case), and the Dominican Republic (1 case). The mean time ± standard deviation between the symptom onset and first sample collection was 3.6 ± 2.1days (range, 0–6 days). The onset of symptoms was defined based on the memory of each patient and it was recorded by physicians Viral RNA was detected in urine samples of all 9 patients (100%), but was detected in serum samples of only 3 out of the 9 patients (33.3%) (Table 1). The viral RNA concentration ranged from 263 to 199,460 copies/mL according to sample type, collection date, and individuals. As for a serological test using an ELISA kit, the acute-phase serum of only 3 of the 9 patients (33.3%) was positive for IgM.

**Table 1:** Laboratory testing results of imported Zika cases in South Korea, from January to July 2016^a^

Patient code	Days (symptom onset to sampling)	qRT-PCR^b^ (copies/mL)		ELISA^c^	
		Serum	Urine	IgM	IgG
1	5 (serum), 6 (urine)	+ (1,347)	+ (39,034)	−	−
2	3	−	+ (762)	−	−
3	3	−	+ (2,313)	−	−
4	2	−	+ (263)	+	−
5	0	+ (588)	+ (199,460)	−	−
6	4	−	+ (3,049)	+	−
7	6	−	+ (24,385)	+	−
8	6	+ (5,326)	+ (934)	−	−
9	IU	NT	+ (6,063)	NT	NT

^a^ *IU* information unavailable, *NT* not tested because specimen was not available, *−* negative, *+* positive

^b^qRT-PCR was performed using genesig Zika virus polyprotein standard kit (PrimerDesign Ltd., UK). Virus titer was estimated by testing pre-quantitated dilution of viral RNA transcripts (provided in the kit) and standard curve calculation generated by the ABI 7500 instrument (Applied Biosystems)

^c^Data were obtained using Anti-Zika Virus IgM/IgG ELISA kits (Euroimmun, Germany)

**Fig. 1:**
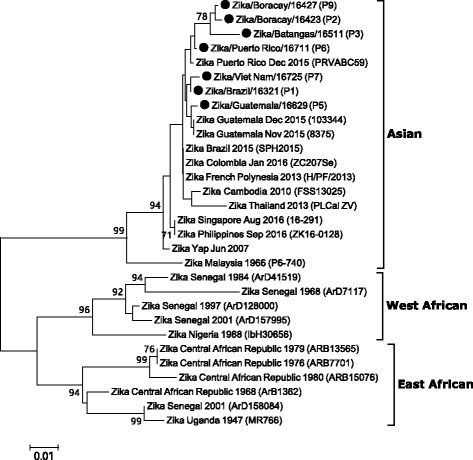
Phylogenetic analysis of the Zika virus based on the partial envelope gene. The tree was constructed by the neighbor-joining method (substitution model: maximum composite likelihood) using MEGA 6 software. The percentage of bootstrap values is shown at each node (1,000 replications). Sequences of Zika viruses detected in this study are indicated as closed circles and the patient code is marked in the parenthesis. Zika virus genotypes are as defined previously [16]

RT-PCR targeting the partial E gene was successfully for 7 of 9 cases (77.8%). The sequence similarities between the seven samples were 97.4–99.7% and 99.1–100% at the nucleotide and amino acid sequence levels, respectively (GenBank accession numbers, KY042039–KY042045). Further, a phylogenetic tree based on the partial E gene sequences obtained from different countries was constructed (Fig. 1). The result indicated that the viruses responsible for infection in the 7 cases belonged to the Asian genotype currently circulating in South America [6].

**Fig. 2:**
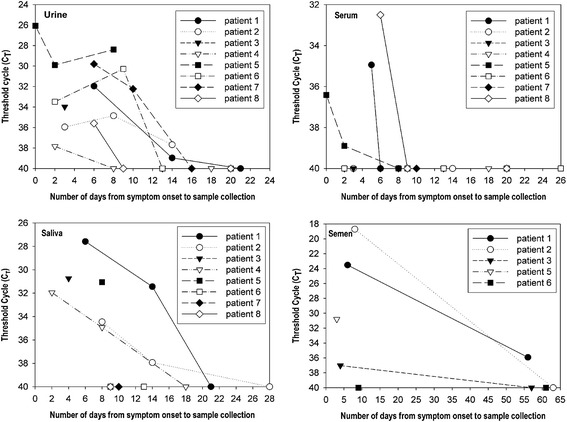
Duration of detectability of Zika viral RNA in different specimens. qRT-PCR was performed using a commercial kit (PrimerDesign Ltd., UK) with serum, urine, saliva, and semen samples. A sample with a threshold cycle (C_T_) number ≤ 40 was considered to be Zika-positive. In patient 3, the final collection of semen was performed at 86th day after the symptom onset and the result was negative. It was not presented for the sake of brevity

Although individual variation was observed and sample numbers were limited, the period over which viral RNA was detectable differed according to the sample type (Fig. 2). Overall, after the day of symptom onset, viral RNA was detectable until 6 days in serum, 14 days in urine and saliva, and 58 days in semen, respectively. In detail, viral RNA was detected only in 5 out of 10 (50%) acute-phase serum specimens (within a week of onset) and the detection rate fell to zero (0/10) in sera collected between 1 and 2 weeks following the symptom onset. However, viral RNA was detectable in all of the 9 (100%) urine samples drawn within a week of onset, and the rate fell to 50% (5/10) in urine drawn between 1 and 2 weeks following the symptom onset. In case of saliva, viral RNA was detected in the 2 samples (100%) drawn within a week of onset, and the detection rate fell to 50% (5/10) in the sample drawn between 1 and 2 weeks following the symptom onset. The results indicate that urine and saliva samples are preferable over serum samples, as they are obtained easily through painless procedures and show higher virus detection rates than those observed in serum samples. Our results are similar with those of previous reports describing the duration of viral RNA detection in different specimens [7–9]. The recovery of Zika virus was reported from urine and saliva as well as from serum and semen [10, 11]. The viruses were isolated from 2 out of 9 patients by inoculating the RT-PCR positive specimens to mammalian cells (BHK-21, Vero, and LLC-MK2); these data will be reported in the near future.

**Fig. 3:**
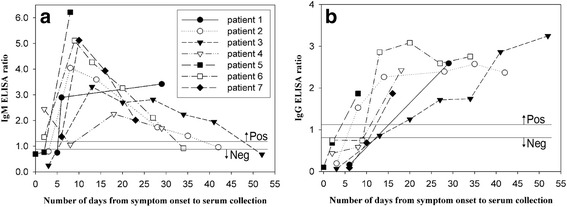
Profile of IgM and IgG responses to Zika virus infection in human serum. ELISA was performed using Anti-Zika Virus IgM/IgG ELISA kits (Euroimmun, Germany). **a** IgM ELISA response, **b** IgG ELISA response; the different symbols represent each patient. Interpretation of the results was based on the ratio of optical density of the patient serum over the value of the calibrator, according to the manufacturer’s instructions. Ratio < 0.8 indicates a negative result, 0.8 ≤ ratio < 1.1 indicates a borderline result, and ratio ≥ 1.1 indicates a positive result

The duration for which antibodies against Zika virus remain detectable in patient’s serum has not yet been established. The humoral immune response to Zika virus after infection was investigated using commercial kits (ELISA and gold rapid test). Anti-Zika Virus IgM and IgG ELISA kits from Euroimmun Inc. (Germany) were successfully used for detection of anti-Zika virus antibodies in 8 of the 9 (88.9%) patients (Fig. 3). However, no positive results were obtained using the Quantitative Human Zika Virus IgM ELISA kit (MyBioSource Inc., Lot E160512AI) and Zika virus IgM/IgG Ab Rapid Test (Biocan Diagnostics Inc., Lot B1815C021616). It could be concluded that the latter two kits were unacceptable for Zika virus testing and require improvement in terms of sensitivity; this was the first evaluation report for the two kits. Using the Euroimmun ELISA kit, IgM against Zika virus was detected as early as 2 days after the symptom onset and IgG was detected from 8 days after the symptom onset (Fig. 3). Of the seven patients whose serum samples were reactive to ELISA and for whom the days of symptom onset available, IgM was detected in acute-phase sera drawn from 4 (57.1%) patients, within 6 days post-onset. However, IgM was positive in all seven patients (100%) from 8 days post-onset. The IgM and IgG were maintained at detectable levels until 41 days and 52 days after the symptom onset, respectively. It is well known that IgM against flaviviruses, including dengue, West Nile, and Japanese encephalitis viruses, as indicated by ELISA, persist for up to several months and up to a year in the case IgG [12–14]. However, it was found that anti-Zika virus IgM antibody titer in patients 2, 3, and 6 fell to equivocal or negative levels at 42, 52, and 34 days post-onset (Fig. 3), respectively. The short duration of antibody detectability in those cases, as indicated by the Euroimmun ELISA, may be caused by the use of nonstructural protein 1 (NS 1) rather than E protein as a diagnostic marker. Further testing using other types of ELISA kits (e.g., ELISA with E protein as target) with a large sample size is required for elucidation of the kinetics of IgM and IgG responses to Zika virus infection in human serum.

The cross-reactivity of anti-Zika virus IgM antibody with other flaviviruses was investigated using flavivirus-specific ELISA kits. IgM cross-reactivity to other flaviviruses was shown in 2 of the 9 (22.2%) confirmed patients. Serum of the patient 2 showed a positive result in West Nile ELISA, and serum of the patient 6 exhibited a positive result in all the ELISAs (West Nile, dengue, and Japanese encephalitis viruses). The results are consistent with those of the previous studies describing the high cross-reactivity in the flavivirus serology [15].

## Conclusions

In conclusion, we reported 9 travel-associated Zika cases in South Korea between January – July 2016. We found that a custom-made qRT-PCR kit and Euroimmun Anti-Zika Virus IgM/IgG ELISA kit were acceptable for routine laboratory testing for Zika virus infection. Additionally, we described virus and antibody kinetics in cases of Zika virus infection using various sample types. These findings should enable diagnostic laboratories to improve their testing programs for Zika virus infection.

## Abbreviations

bp: Base pair
C_T_: Threshold cycle
E: Envelope
ELISA: Enzyme-Linked Immunosorbent Assay
Ig: Immunoglobulin
KCDC: Korea Centers for Disease Control and Prevention
NS1: Nonstructural protein 1
RT-PCR: Reverse transcription-polymerase chain reaction
